# Chimeric Mice Engrafted With Canine Hepatocytes Exhibits Similar AAV Transduction Efficiency to Hemophilia B Dog

**DOI:** 10.3389/fphar.2022.815317

**Published:** 2022-01-31

**Authors:** Wenwei Shao, Junjiang Sun, Xiaojing Chen, Amanda Dobbins, Elizabeth P. Merricks, R. Jude Samulski, Timothy C. Nichols, Chengwen Li

**Affiliations:** ^1^ Academy of Medical Engineering and Translational Medicine, Tianjin University, Tianjin, China; ^2^ Gene Therapy Center, University of North Carolina at Chapel Hill, Chapel Hill, NC, United States; ^3^ Division of Pharmacoengineering and Molecular Pharmaceutics, Eshelman School of Pharmacy, University of North Carolina at Chapel Hill, Chapel Hill, NC, United States; ^4^ Department of Pathology and Laboratory Medicine and The Blood Research Center, University of North Carolina at Chapel Hill, Chapel Hill, NC, United States; ^5^ Department of Pharmacology, School of Medicine, University of North Carolina at Chapel Hill, Chapel Hill, NC, United States; ^6^ Department of Pediatrics, University of North Carolina at Chapel Hill, Chapel Hill, NC, United States; ^7^ Carolina Institute for Developmental Disabilities, University of North Carolina at Chapel Hill, Chapel Hill, NC, United States

**Keywords:** liver transduction, chimeric mice, hemophilia B, canine hepatocytes, AAV (Adeno-associated virus)

## Abstract

Adeno-associated virus (AAV) mediated gene therapy has been successfully applied in clinical trials, including hemophilia. Novel AAV vectors have been developed with enhanced transduction and specific tissue tropism. Considering the difference in efficacy of AAV transduction between animal models and patients, the chimeric xenograft mouse model with human hepatocytes has unique advantages of studying AAV transduction efficiency in human hepatocytes. However, it is unclear whether the results in humanized mice can predict AAV transduction efficiency in human hepatocytes. To address this issue, we studied the AAV transduction efficacy in canine hepatocytes in both canine hepatocyte xenografted mice and real dogs. After administration of AAV vectors from different serotypes into canine hepatocyte xenograft mice, AAV8 induced the best canine hepatocyte transduction followed by AAV9, then AAV3, 7, 5 and 2. After administration of AAV/cFIX (cFIX-opt-R338L) vectors in hemophilia B dogs, consistent with the result in chimeric mice, AAV8 induced the highest cFIX protein expression and function, followed by AAV9 and then AAV2. These results suggest that mice xenografted with hepatocytes from different species could be used to predict the AAV liver transduction in real species and highlight this potential platform to explore novel AAV variants for future clinical applications.

## Introduction

Adeno-associated virus (AAV) has been employed in hundreds of clinical trials worldwide as an *in vivo* gene therapy vector due to its unique beneficial properties including long-term transgene expression and low immunogenicity. To cure a monogenic disease, a reasonable AAV serotype should be chosen for a specific tropism and precise targeting effect *in vivo*. A number of distinct AAV serotypes (AAV1-AAV13) and over 100 variants have been isolated (Wu et al., 2006; Schmidt et al., 2008), meanwhile a branch of mutants has been developed through rational design or directed evolution using an AAV shuffling library (Grimm and Büning, 2017; Lee et al., 2018; Domenger and Grimm, 2019; Pei et al., 2020), aiming to gain specific tropism or escape immune elimination (Bartel et al., 2012; Kanaan et al., 2017; Chai et al., 2018; Pei et al., 2020). It has been demonstrated that AAV serotypes 1–6 have a relatively high transduction efficiency *in vitro*, while AAV serotypes 7–13 are able to induce a high transduction *in vivo* and a poor transduction *in vitro* (Wu et al., 2006; Srivastava, 2016). However, in humans, there is a lack of direct evidence to verify the specific targeting capabilities of AAV serotypes due to the limited number of patients involved in clinical trials and the related ethical constraints.

In recent years, gene therapy for hemophilia A/B, which are directly caused by mutations in coagulation factor VIII/IX (Furie and Furie, 1988; Giannelli and Green, 1996), has shown promising results. Different AAV serotypes [AAV2 (Manno et al., 2006), AAV5 (Rangarajan et al., 2017), AAV8 (Nathwani et al., 2014)] and variants/mutants (AAVrh10 (NCT02618915) with liver tropism in mouse and non-human primate models have been used in clinical trials in patients with hemophilia. In the first successful AAV-based gene therapy study for hemophilia (NCT00979238), recombinant AAV8 encoding codon-optimized FIX was administered to 10 hemophilia B patients. Persistent FIX levels (1–6%) were observed in all individuals with varying doses (2 × 10^11^, 6 × 10^11^ and 2 × 10^12^ vg/kg) and a stable level around 5% has persisted for over 7 years follow-up periods for the high dose cohort (Nathwani et al., 2011a; Nathwani et al., 2014; Nathwani, 2019), resulting in a reduction in spontaneous bleeding and FIX protein usage. The liver toxicity induced by AAV administration was reported as mild with a transient increase of ALT and AST (Manno et al., 2006). Overall, AAVs have been well-recognized as an ideal *in vivo* gene carrier to cure hemophilia and other disorders.

From bench research to the bedside, animal models are critical to test the transduction efficiency and side effects of AAV vectors *in vivo*. Mouse models were widely used in AAV related *in vivo* studies. However, the transduction efficiency results derived from the mouse models did not translate well to clinical trials (Hurlbut et al., 2010). When AAV8 vectors encoding coagulation factor IX driven by the liver specific promoter in a self-complementary (scAAV8/hFIX) format were administered in a hemophilia murine model, a high transduction efficiency and improved hemostasis were observed even though a lower dose of vectors was used. However, a ten-fold higher dose of scAAV8 vectors in rhesus macaque model (Nathwani et al., 2006; Nathwani et al., 2007; Nathwani et al., 2011b) and even over 100-fold dose in clinical trials were needed to achieve a similar therapeutic level to that in mice (Nathwani et al., 2011b). AAV8 has been reported to have a higher transduction efficiency in the liver than AAV2 both in the murine and non-human primate models (Nathwani et al., 2006; Nietupski et al., 2011). However, as revealed by clinical trials, AAV2 and AAV8 showed a similar gene therapy effect in hemophilia patients (Nathwani et al., 2014). These findings indicate the discrepancies of AAV transduction between different species, and results generated from animal models may not be directly translated into clinical trials, which highlights the urgent need to establish alternative models to test and develop novel AAV vectors for future clinical trials.

Non-human primates are widely considered to be the ideal model in preclinical trials. However, due to the unavailability of a disease model in non-human primates, other large animal models, such as dog, play an important role and have commonly been used in the translational studies from preclinical rodents to human clinical trials. Large animals can be more predictive of clinical studies compared to mice due to longer life expectancies allowing for more thorough follow-ups. Indeed, hemophilia A/B canine models have been used for long-term follow-up for AAV gene therapy (Sun et al., 2018; Nguyen et al., 2021). More importantly, large animals have strong similarities to humans, such as their immunogenicity profiles (Ziegler et al., 2016; Graham et al., 2020). Hemophilia A/B canine models have been well established to study the pathophysiology of AAV gene therapy, and the results are readily translated into clinical application (White et al., 1997; Brinkhous et al., 2002).

The chimeric mouse model has its unique advantage for studying AAV tropism, which could provide direct evidence of AAVs’ transduction efficiency to specific cells *in vivo*. For AAV serotype selection or AAV evolution, the human hepatocyte chimeric murine model has been established and adopted in several studies. Herzog et al., reported that AAV3, AAV5, AAV8, and AAV9 induced a higher transduction efficiency in mouse hepatocytes than human hepatocytes in a humanized mouse model, which indicated substantial differences for AAV vectors to transduce murine and human hepatocytes *in vivo* (Vercauteren et al., 2016). Our previous study revealed that AAV7 had a higher transduction efficiency than other serotypes in human hepatocytes in chimeric mice (Shao et al., 2019). The chimeric xenograft mouse model with human hepatocytes may represent a more precise platform for both selecting and evaluating clinically relevant rAAV serotypes for human gene therapeutic applications (Shao et al., 2019). Nonetheless, it still remains unclear whether the transduction efficiency from AAV vectors in the chimeric mouse model is able to precisely predict the result in humans. To address this issue, in this study, we used chimeric mice xenografted with canine hepatocytes and studied the transduction efficiency in canine hepatocytes in both chimeric mice and hemophilia B dogs. We first compared the transduction efficiency of different AAV serotypes in the canine hepatocyte xenograft mouse model. Next, we investigated the transgene FIX expression, the immunogenicity, and liver injury after liver targeting from different AAV serotypes in hemophilia B dogs. The results indicate that chimeric xenograft mice with hepatocytes from other species could indeed be used to predict the liver transduction efficiency from AAV vectors in those species.

## Materials and Methods

### Cell Line

HEK-293 cells were grown in Dulbecco’s Modified Eagle’s Medium with 10% FBS and 1% penicillin–streptomycin at 37°C in 5% CO_2_. It was negative for the *mycoplasma* contamination test.

### AAV Vector Production and Purification

AAV virus was produced using the triple plasmid transfection system as previously described (Xiao et al., 1998). Briefly, HEK-293 cells were transfected with an AAV transgene plasmid (scpTR-CBh-GFP, scpTR-TTR-cFΙX, or sspTR-CBA-Luciferase), an AAV helper plasmid with Rep and Cap genes, and the adenovirus helper plasmid pXX6-80. Cells were harvested and lysed 48 h post-transfection. AAV vectors were purified by cesium chloride (CsCl) gradient ultracentrifugation. The virus titer was determined by Q-PCR with ITR primers and transgene specific primers. The AAV titers and genome integrity were also confirmed by alkaline gel.

### Animals

All procedures performed on animals and care were approved by the University of North Carolina Institutional Animal Care and Use Committee. FRG (Fah^−/−^, Rag2^−/−^ and Ilr2g^−/−^) mice were purchased from Yecuris (Oregon, United States) and maintained in a sterile environment with Nitisinone (NTBC) in drinking water (16 mg/L). FIX^−/−^ hemophilia mice were originally supplied by Dr. Darrel Stafford and bred in house (Lin et al., 1997). All the mice, including FRG mice, were maintained in a specific pathogen-free facility at the University of North Carolina at Chapel Hill in accordance with the NIH guidelines.

Six hemophilia B dogs (O02, S16, Alex, Ralphie, P43 and P44) were housed at the Francis Owen Blood Research Laboratory at the University of North Carolina at Chapel Hill, NC, which is a US Department of Agriculture approved facility as described before (Monahan et al., 2010).

### Generation of Canine Hepatocyte Xenograft Mouse Model

FRG mice were engrafted with canine hepatocytes as described previously (Azuma et al., 2007). Briefly, FRG mice were injected intravenously with 5 × 10^9^ pfu Ad virus encoding the gene of the secreted form of human urokinase a day before the xenograft experiment. 5 × 10^9^ viable canine hepatocytes (Triangle Research Labs of Lonza Group) in 100 μl DMEM were injected intrasplenically. The concentration of NTBC was gradually decreased to 0 within 1 week (1.6 mg/L, day 0–2; 0.8 mg/L, day 3–4; 0.4 mg/L, day 5–6; 0 mg/L, day 7) after transplantation. Two weeks after NTBC withdrawal, mice were treated with the drug for another 5 days and then taken off permanently. To verify the establishment of chimeric mice xenografted with canine hepatocytes, we detected the concentration of canine albumin in blood by ELISA (Abcam, Waltham, MA, United States) at week 9 after injection of canine hepatocytes. The surviving chimeric mice had serum albumin levels ranging from 7 to 21 g/L (30- approximately 80% of normal level in dogs, Supplementary Figure S1).

Canine hepatocytes were supplied by 2 separate donors. The mice successfully xenografted with canine hepatocytes were used for AAV transduction experiments. The comparisons of AAV transduction efficiency between different serotypes was performed with xenografted hepatocytes from the same donor.

### Animal Treatment

Dogs were treated by AAV/canine factor IX (cFIX) at a dose of 1×10^12^/kg via portal vein administration. 1 × 10^11^ (for chimeric mice) or 1 × 10^10^ particles (for FIX^−/−^ hemophilia mice) of AAV vectors in 100 μl PBS were administered *via* retro-orbital vein injection for each mouse.

### Immunostaining

Mice livers were harvested and stained as described before (Shao et al., 2019). Briefly, liver sections were incubated with goat anti-canine albumin (Bethyl) overnight at 4°C in a humidified chamber. After washing with PBS, the sections were incubated with the secondary antibody, anti-goat Alexa Flour 488-conjugated secondary antibodies (Invitrogen), at 20°C for 2 h s. After washing, the sections were blocked again and then stained with chicken anti-GFP antibody (Aves labs) at 4°C overnight, followed by anti-chicken Alexa Flour 594-conjugated secondary antibody (Molecular Probes) staining. Finally, sections were stained with DAPI and mounted on glass slides. Mean and standard deviation were determined from five liver IF pictures for each mouse.

### Positive Staining Cell Counting

After immunostaining, we used images to count and quantify the positive staining cells. First, we selected at least five areas under the microscope randomly from captured images. Then the single cell staining condition was scored as negative, low, medium, or high staining according to the fluorescence density. Medium and high staining cells were counted as positive cells. ImageJ was used to count positively labeled green staining hepatocytes. Double positive cells shown as golden were counted manually. Three individual investigators were assigned to perform the counting blindly. Representative low, medium, or high staining cells for GFP or canine albumin were arrowed and the cells showing co-localization of canine albumin and GFP were indicated (Supplementary Figure S6).

### Flow Cytometry

To detect regulatory T cells, PBMCs isolated from the canine peripheral blood were stained with a PE-CD25 antibody (eBioscience) and a PE/Cy7-CD4 antibody (eBioscience) for 30 min. Then the cells were fixed and permeabilized for Foxp3 Transcription Factor Staining following the manufacturer’s recommendation (Thermo Fisher Scientific). Next, the PBMCs were stained with PE/Cy5.5-conjugated Foxp3 antibody (eBioscience) and then analyzed by the Beckman Coulter CyAn ADP.

### FIX Specific ELISA and FIX Activity in Dog and Mice

Anti-coagulated blood samples were obtained from normal canine control, the untreated hemophilia B dog, and treated hemophilia B dogs or mice as described previously (Du et al., 2013). Canine FIX (cFIX) protein level in blood was measured by the Canine Factor IX Paired Antibody Set from Affinity Biological (ON, Canada). The standard curve of cFIX was generated based on serial dilution of normal canine plasma (100%) with pooled hemophilia dog or mouse plasma. FIX activity was measured by the one-stage FIX activity assay (FIX-specific aPTT) using the STart four coagulation analyzer (Diagnostica Stago) as described (Schuettrumpf et al., 2005). FIX activity was generated based on the standard curve by diluting the recombinant FIX of BeneFIX^®^ (Pfizer, Philadelphia, PA) with pooled hemophilia dog or mouse plasma. Recombinant FIX diluted to 1 unit/ml was used as 100% activity.

### WBCT Assay

WBCT was performed using whole-blood samples, as previously described (Du et al., 2013). WBCT is a modification of the Lee–White clotting time using two siliconized glass tubes (Becton Dickinson, Rutherford, NJ) at 28°C. 1 ml whole blood was drawn and 0.5 ml blood was distributed into each tube. After 1 min, one tube was tilted every 30 s, while the other was left undisturbed. When a clot formed in the tilted tube, the second tube was then tilted every 30 s until a clot formed. The time for formation of a fully gelled clot in the second tube was recorded as the WBCT.

### Liver Enzyme Analysis

Serum alanine aminotransferase (ALT) and aspartate transaminase (AST) levels were detected at the Histopathology Facility at UNC at Chapel Hill. Serum chemistry tests for dog samples were performed at ANTECH Diagnostics (Chapel Hill, NC, United States).

### Neutralizing Antibody Analysis

1 × 10^5^ Huh7 cells were seeded in a well of a 48-well plate and transduced with AAV-Luciferase vectors pre-incubated with plasma at a ratio of 1,000 particles/cell. After 48 h, cells were lysed and luciferase activity was measured with a Wallac-1420 Victor two automated plate reader. The IC50 of the NAb was defined as the highest dilution that reduced luciferase activity to 50% of that in cells transduced with AAV-Luciferase vectors incubated with PBS as the control. AAV vectors were pre-incubated with serial dilutions of canine plasma for 2 h at 4°C. First, the rough NAb range was obtained using a 10-fold dilution series, with undiluted plasma as the starting point. Then a two-fold dilution within the NAb range was used to determine the accurate NAb titer.

### Statistical Analysis

All statistical calculations were performed using GraphPad Prism 7.0. Data are presented as mean ± SD. Data from single comparisons were evaluated by the paired Student’s t test. 2-way ANOVA was used for multiple group comparisons. Differences were considered statistically significant when *p* < .05.

## Results

### AAV8 Induced Higher Canine Hepatocyte Transduction in Chimeric Mice Xenografted With Canine Hepatocytes

To investigate the transduction efficiency of AAV vectors in canine hepatocytes, we first established a chimeric mouse xenograft model with canine hepatocytes. One million fresh canine hepatocytes were injected intrasplenically into FRG mice, followed by NTBC withdrawal over the next 5 days 2 weeks after NTBC withdrawal, the mice were treated with the drug for another 5 days and then taken off permanently. Thereafter, xenograft mice with canine hepatocytes repopulation was established. To verify the establishment of chimeric mice xengrafted with canine hepatocytes, the concentration of canine albumin in blood was determined by ELISA at week 9 after injection of canine hepatocytes. The canine albumin level in xenograft mice serum ranged from 7 g/L to 21 g/L (Supplementary Figure S1), corresponding to 30% to approximately 80% engraft efficiency, according to previous studies (Azuma et al., 2007; Billerbeck et al., 2016).

**FIGURE 1:**
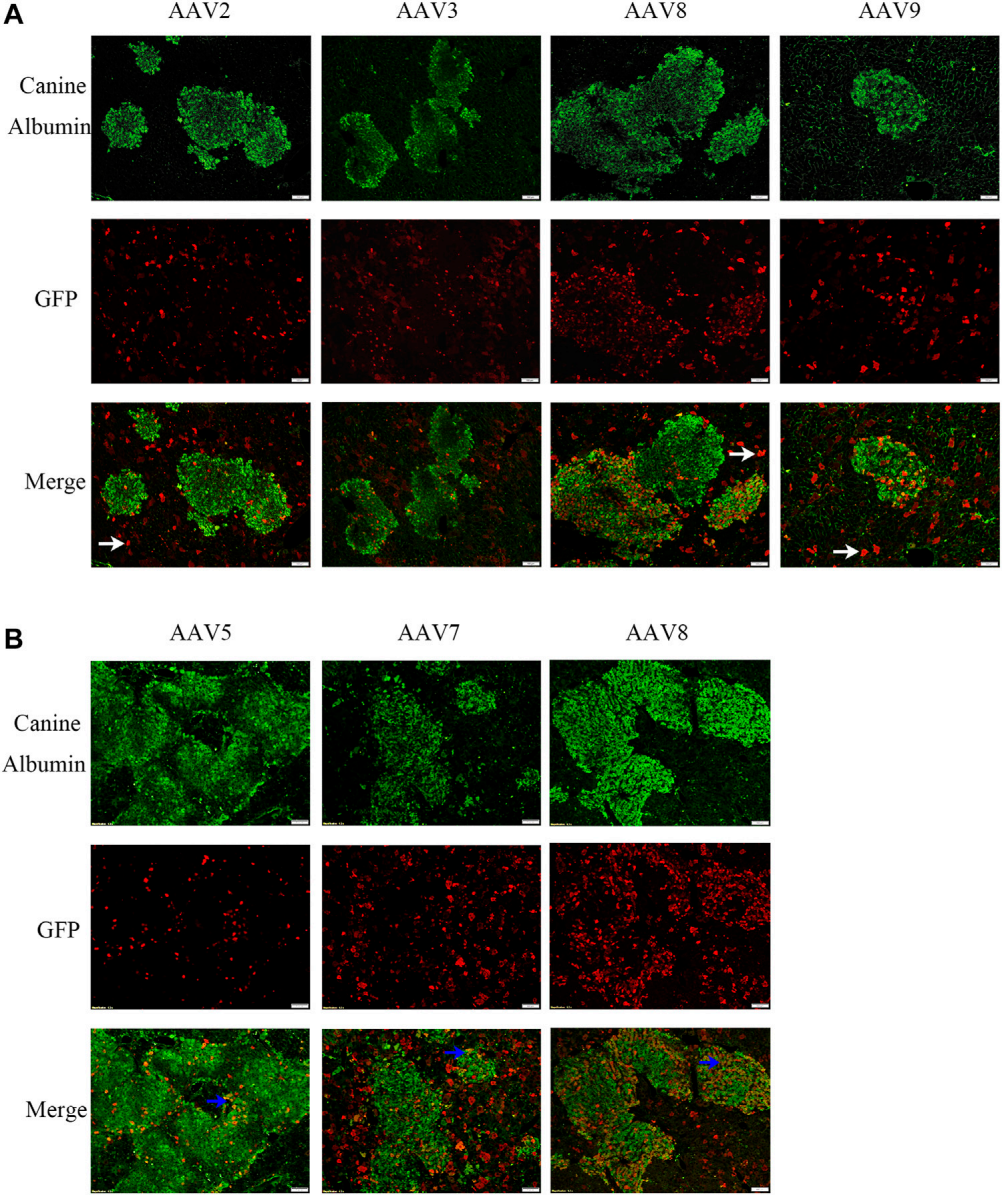
AAV transduction efficiency of canine hepatocytes in canine xenograft mice. AAV vectors of varying serotypes packaged with the GFP transgene were administered in canine hepatocyte xenograft mice at a dose of 1×10^11^vg/mouse. Four weeks later, the livers were harvested. GFP (red) expression in canine hepatocytes (green) was detected by immunofluorescence. The scale represented 100 μm. **(A)** AAV2, AAV3, AAV8 or AAV9 vectors were injected into canine hepatocyte xenograft mice. Hepatocytes were from the same source. **(B)** AAV5, AAV7 or AAV8 vectors were injected into canine hepatocyte xenograft mice. Hepatocytes were again from the same source. The representative murine hepatocytes which were transduced were indicated by white arrows, while transduced canine hepatocytes were indicated by blue arrows.

Several AAV serotypes were used to screen canine hepatocyte transduction in the xenograft mice. In the first experiment, AAV vectors from serotypes 2, 3, 8, and 9 were tested in mice xenografted with hepatocytes from canine donor #1. 4 weeks after systemic injection of 1×10^11^ vg/mouse of scAAV-GFP vectors, the mice were sacrificed and the livers were collected for transduction analysis in canine hepatocytes. The AAV transduction efficiency was analyzed by double staining of canine albumin and GFP. Wild type canine liver and mouse liver were used as positive and negative controls (Supplementary Figure S2). As shown in Figure 1A and Table 1, AAV8 induced significantly better canine hepatocyte transduction (12.65%, 11.49% and 11.43% in three individual mice), followed by AAV9 (8.57%), then AAV3 (6.74% and 4.72%) and AAV2 (2.76% and 3.76%). To extend the study to other AAV serotypes, we conducted a second experiment, where we tested AAV5, AAV7 and AAV8. The same dose of scAAV-GFP vectors were injected into mice engrafted with donor #2 canine hepatocytes. As shown in Figure 1B and Table 1, AAV8 still showed the highest transduction efficiency (25.71% and 30.73%), followed by AAV7 (5.73% and 9.41%), and AAV5 (11.78% and 2.15%). Combining these results from both experiments, we conclude that AAV8 has shown the highest transduction efficiency in canine hepatocytes in chimeric mice.

**TABLE 1:** AAV vector transduction efficiency of canine hepatocytes in canine xenograft mice.

1st			2nd		
Mouse ID	AAV	Transduction % (mean ± SD)*	Mouse ID	AAV	Transduction % (mean ± SD)*
260	2	2.76 ± 0.47	703	5	11.78 ± 1.91
268	2	3.76 ± 1.61	704	5	2.15 ± 0.21
251	3	6.74 ± 1.51	705	7	5.73 ± 0.76
254	3	4.72 ± 1.28	707	7	9.41 ± 1.39
262	8	12.65 ± 4.77	709	8	25.71 ± 3.08
278	8	11.49 ± 1.91	710	8	30.73 ± 5.52
281	8	11.43 ± 4.55			
287	9	8.57 ± 5.48			

### Optimization of Canine FIX Transgene in Hemophilia B Mice

**FIGURE 2:**
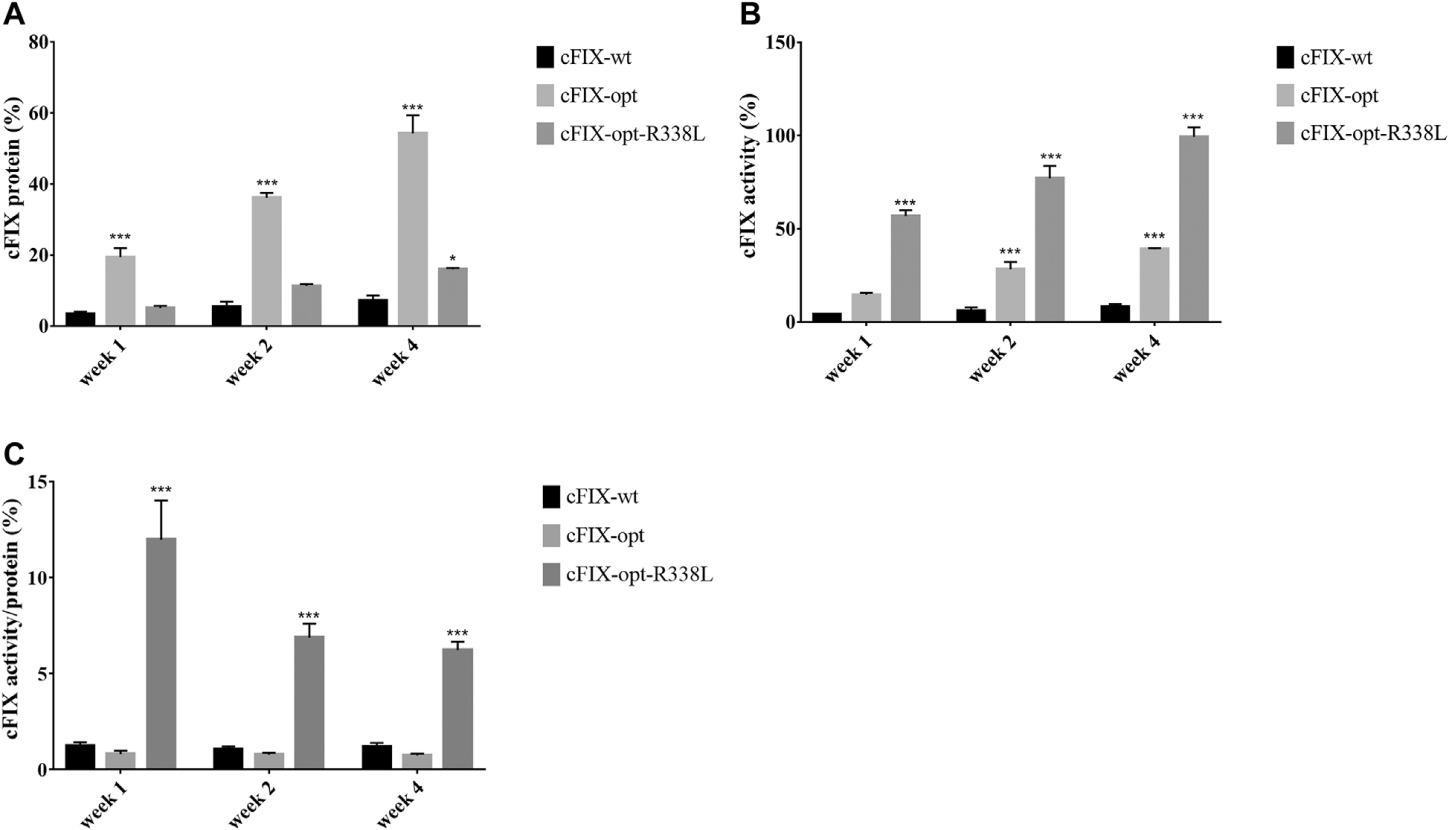
Examine optimized cFIX mutant cassette in hemophilia B mice. Mice with FIX deficiency received 1 × 10^10^ particles of scAAV8/cFIX-wt, scAAV8/cFIX-opt or scAAV8/cFIX-opt-R338L vectors (*n* = 4). Blood was collected from the retro-orbital venous plexus at weeks 1, two and 4. **(A)** canine FIX concentrations in plasma were detected by ELISA. **(B)** cFIX activities were measured by aPTT assay. The ratio of cFIX protein to activity was calculated and shown in **(C)** ***p < .001, *p < .05.

To study whether the results for canine hepatocyte transduction in xenograft mice with canine hepatocytes predict the actual transduction efficiency in the liver of hemophilia B dogs, we first designed a codon-optimized canine FIX cassette with R338L mutation driven by the TTR promoter in a self-complementary (sc) AAV vector. It has been demonstrated that optimization of the human FIX transgene is able to increase hFIX expression, while introduction of the point mutation R338L in hFIX enhances hFIX activity. Therefore, we made three different canine FIX cassettes: the wild type cFIX (cFIX-wt), codon-optimized cFIX (cFIX-opt), and optimized cFIX with R338L mutant (cFIX-opt-R338L). These cassettes were packaged into AAV8 virions to explore cFIX expression and activity. 1 × 10^10^ particles of AAV8 vectors were administered into hemophilia B mice. Blood was collected at the indicated time points for cFIX expression and activity analysis. Compared to the wild type cassette, cFIX-opt exhibited significantly higher expression levels, while cFIX-opt-R338L showed a similar level of cFIX expression to cFIX-wt (Figure 2A). Factor IX activity in the cFIX-opt-R338L group was significantly higher than that in the cFIX-opt and cFIX-wt groups (Figure 2B). The ratio of activity to protein demonstrated that cFIX-opt-R338L exhibited a six- to 10-fold higher specific activity of the protein throughout the 4 weeks (Figure 2C), which was consistent with previous reports for human FIX with the R338L mutation (Finn et al., 2012; Crudele et al., 2015).

**FIGURE 3:**
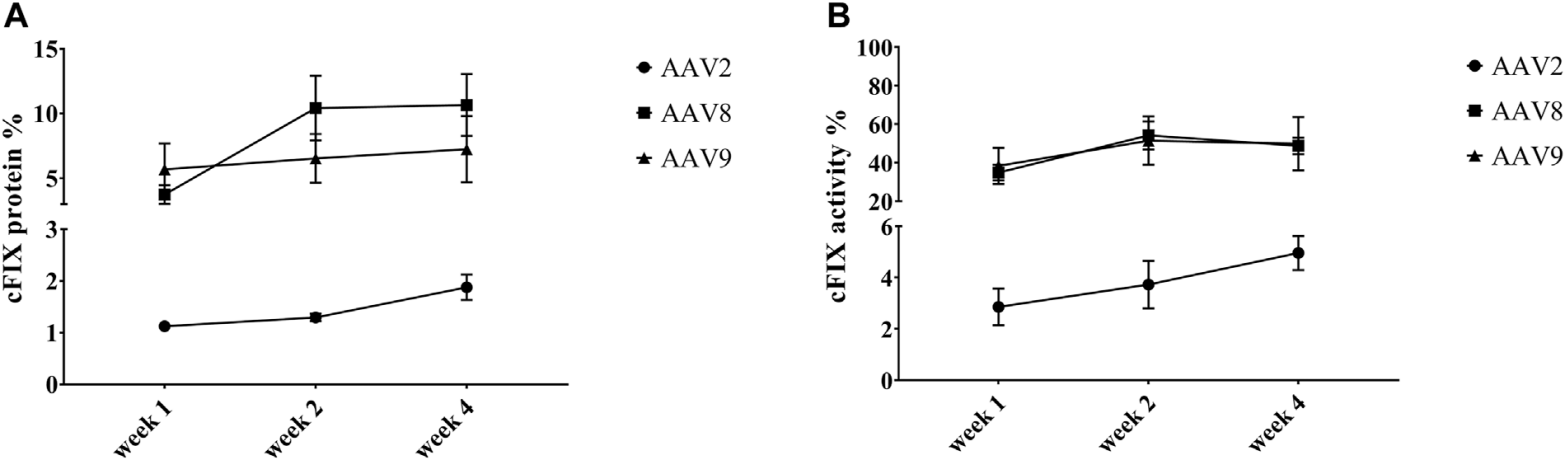
Expression and activity of cFIX in hemophilia B mice after administration of AAV2, AAV8 or AAV9 vectors. Mice with FIX deficiency received 1 × 10^10^ particles of AAV2, AAV8 or AAV9 with cFIX-opt-R338L transgene (*n* = 5). Blood was collected from the retro-orbital venous plexus at weeks 1, 2 and 4. **(A)** canine FIX concentrations in plasmas were detected by ELISA. **(B)** cFIX activities were measured by aPTT assay.

To explore the transduction efficiency of different AAV serotypes in the liver of hemophilia B mice, the cFIX-opt-R338L construct was packaged into AAV2, AAV8 and AAV9 vectors since these serotypes had various transduction efficiency in canine hepatocytes of chimeric mice and have been studied in clinical trials for human liver targeting. 1 × 10^10^ particles of AAV vectors were injected into hemophilia B mice via the retro-orbital vein. We examined the expression and activity of cFIX at weeks 1, 2, and 4 post-AAV administration of AAV2, AAV8 or AAV9 vectors. AAV2 induced much lower cFIX expression in blood than AAV8 and AAV9, while no difference in cFIX expression was demonstrated between AAV8 and AAV9 (Figure 3A). Consistent with cFIX expression, cFIX activity was higher in mice treated with AAV8 and AAV9 than that with AAV2, and similar cFIX activity was observed with AAV8 and AAV9 (Figure 3B).

### Evaluation of AAV2, AAV8, and AAV9 Liver Transduction Efficiency With Canine FIX Transgene in Hemophilia B Dog

**FIGURE 4:**
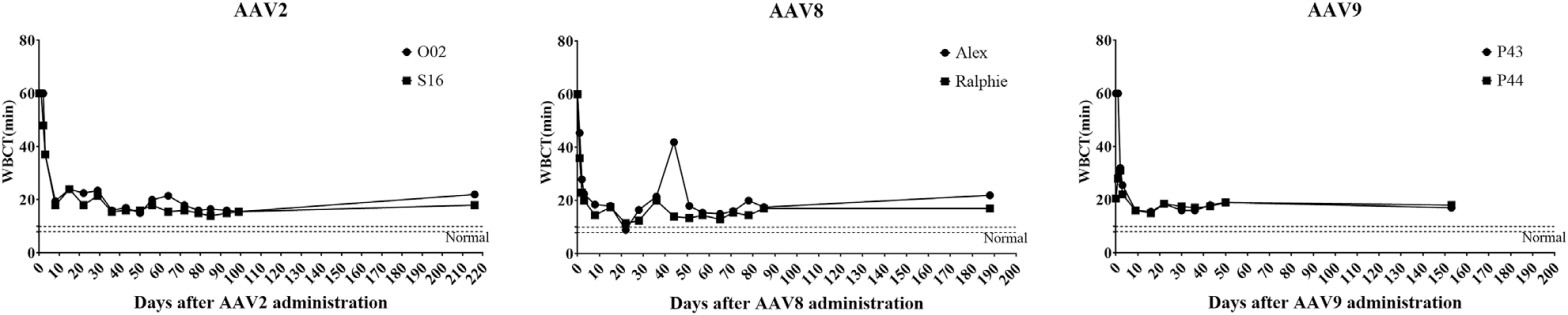
Whole-blood clotting time (WBCT) in hemophilia B dog after administration of AAV2, AAV8 or AAV9 vectors. Hemophilia B dogs received 1 × 10^12^vg/kg of AAV2, AAV8 or AAV9 packaged with a cFIX-opt-R338L transgene. Blood was collected at different timepoints. The baseline of WBCT in hemophilic dogs is over 20 min. A range of 6–12 min in normal dogs has been indicated in the box region as normal.

Next, hemophilia B dogs were administered with 1 × 10^12^ particles/kg of AAV vectors encoding the cFIX-opt-R338L transgene. WBCT was measured over 153 days to evaluate the correction of the plasma clotting potential. In hemostatically normal dogs, the WBCT is 6–12 min; the WBCT is > at least 20 min in naïve hemophilia dogs and most often over 60 min. Within 3 days after systemic injection of AAV8 or AAV9 vectors via peripheral veins, the WBCT decreased to normal or close to normal (Figure 4). It took 8 days to decrease the WBCT to near normal level (<20) in hemophilia B dogs treated with AAV2 vector (Figure 4). The improvement of WBCT in all treated hemophilia B dogs persisted during the observation periods. Thromboelastography (TEG) assay was also performed to quantify the correction of the clotting potential after AAV vector administration (Supplementary Figure S3) and showed improved hemostasis in all hemophilia B dogs treated with AAV vectors from these three serotypes.

**FIGURE 5:**
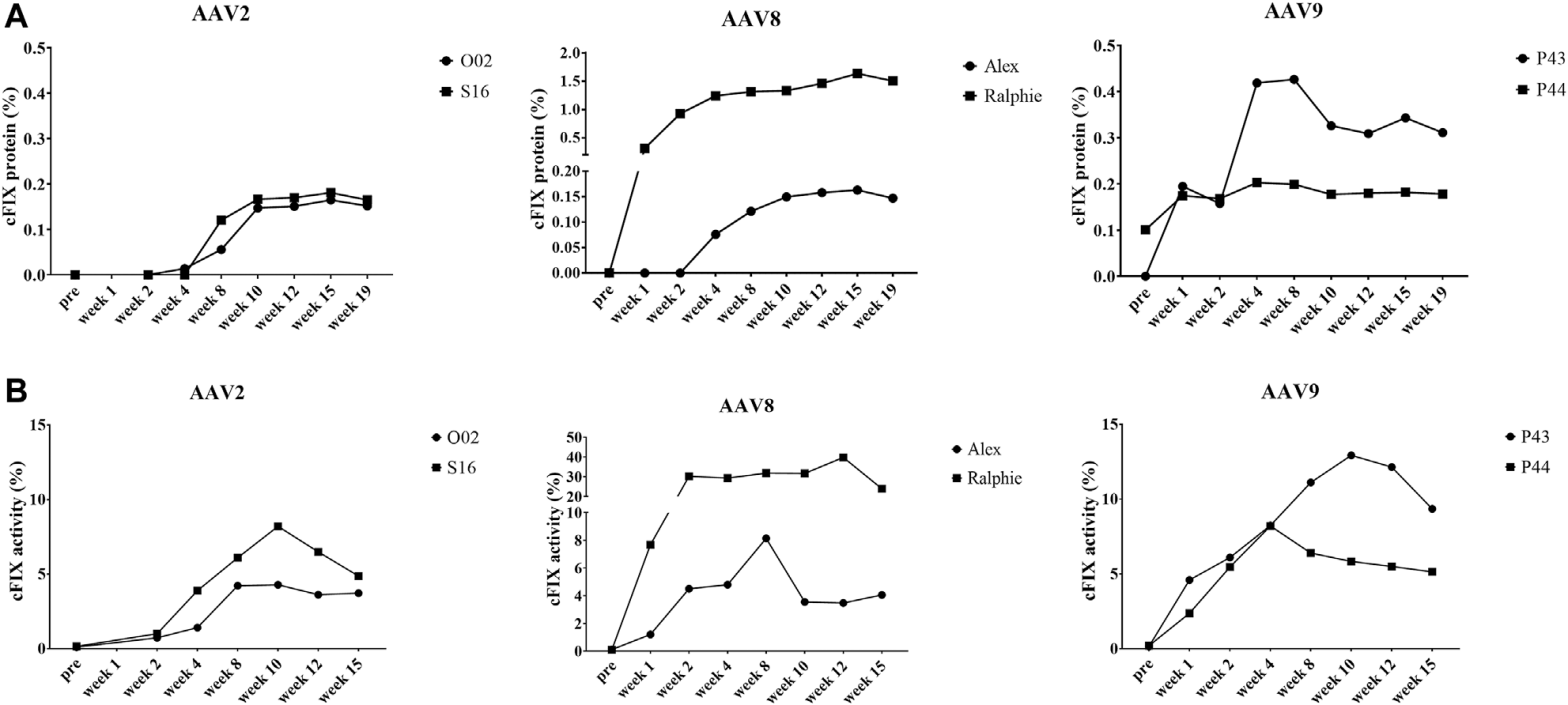
Expression and activity of cFIX in hemophilia B dog after administration of AAV2, AAV8 or AAV9 vectors. Hemophilia B dogs received 1 × 10^12^vg/kg of AAV2, AAV8 or AAV9 packaged with a cFIX-opt-R338L transgene. Blood was collected at different timepoints. **(A)** canine FIX concentrations in plasmas were detected by ELISA. **(B)** cFIX activities were measured by aPTT assay.

We also detected the expression and activity of cFIX in the circulating blood of hemophilia B dogs treated with AAV2, AAV8, and AAV9 vectors encoding cFIX-opt-R338L. Administration of AAV2 vectors induced lower transgene cFIX expression (<0.2%) and clotting activity (<10%) in both hemophilia dogs (Figure 5). Similar cFIX protein levels and activity were observed in one dog from each AAV8 (Alex) and AAV9 group (P44). In the AAV8 group, another dog, Ralphie, exhibited the highest protein levels and clotting activity among all six dogs. A dog that received the AAV9 vector administration, P43, showed higher cFIX expression levels and activity than AAV2 (Figure 5). Generally, these data indicate AAV8 was the best at transducing hepatocytes in hemophilia B dogs among the three AAV serotypes, followed by AAV9. The pattern for AAV liver transduction in hemophilia B dogs was similar to that in chimeric mice xenografted with canine hepatocytes in contrast to the results in hemophilia mice, in which AAV8 and AAV9 transduced canine hepatocytes with similar efficiency (Figure 3). Collectively, these results suggest that transduction efficiency in canine hepatocytes from chimeric mouse model may be able to predict the actual transduction potential in the liver of hemophilia B dogs.

### Immune Response Induced by AAV Vectors in Hemophilia B Dogs After AAV2-, AAV8- and AAV9-cFIX Administration

AAV capsid specific cytotoxic T lymphocyte (CTLs) mediated elimination of AAV transduced hepatocytes has been suggested to contribute to therapeutic failure in hemophilia clinical trials. We studied whether liver toxicity would be induced in hemophilia dogs after AAV administration by measurement of liver enzymes as the biomarker. There was no substantial change of alanine transaminase (ALT) and aspartate transaminase (AST) levels in the circulating blood of hemophilia dogs after AAV administration (Supplementary Figure S4). We also analyzed the capsid specific CTL response after AAV administration. Compared to control without capsid peptide pulse, no significant increase (over 50% of control) of capsid specific T cell proliferation with capsid peptide stimulation was observed in any hemophilia B dogs treated with AAV vectors (Supplementary Figure S5).

**FIGURE 6:**
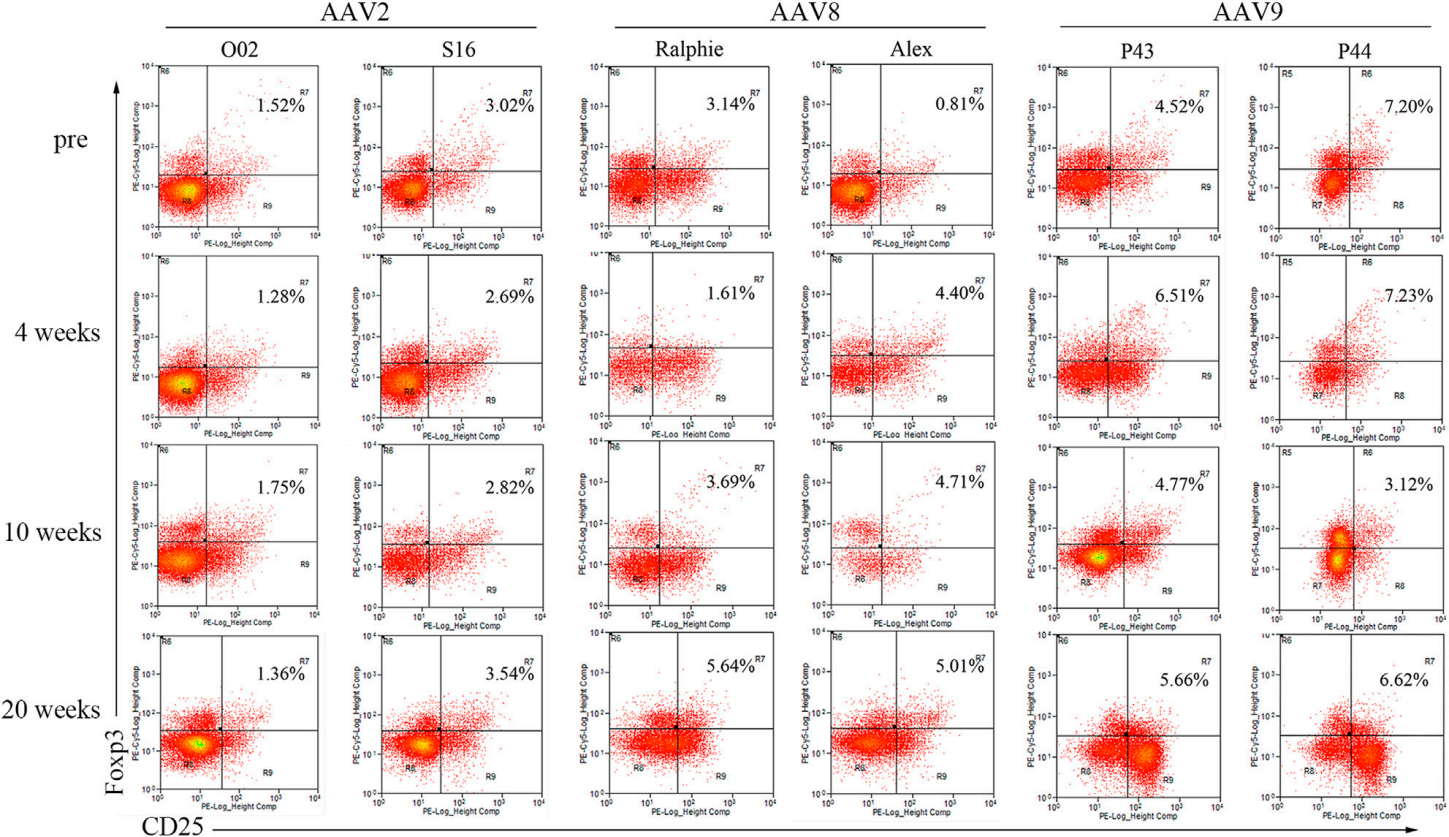
The profile of T regulatory cells in canine PBMCs after administration of varying AAV vector serotypes. PBMCs, separated from the whole blood of hemophilia dogs that were injected with AAV2, AAV8 or AAV9 vectors at a dose of 1 × 10^12^vg/kg, welacbre stained with CD4, CD25 and Foxp3 antibodies, and then analyzed by Flow Cytometry. The percentage of Treg cells (CD4^+^ CD25^+^ Foxp3^+^) was shown.

It has been demonstrated that Treg is associated with immune tolerance induced by AAV liver targeting. Therefore, we detected T regulatory cells (Treg) in the blood of the hemophilia dogs after AAV administration. For AAV2 and AAV9, a change of the Treg percentage in the blood was not observed between pre- and post-AAV injection (less than 20%, Figure 6). Interestingly, Tregs were dramatically altered after AAV8 administration. In dogs Ralphie and Alex, the Treg number increased from 3.14% to 0.81% pre-injection to 5.64% and 5.01% at week 20 after AAV8 administration, respectively (Figure 6).

**FIGURE 7:**
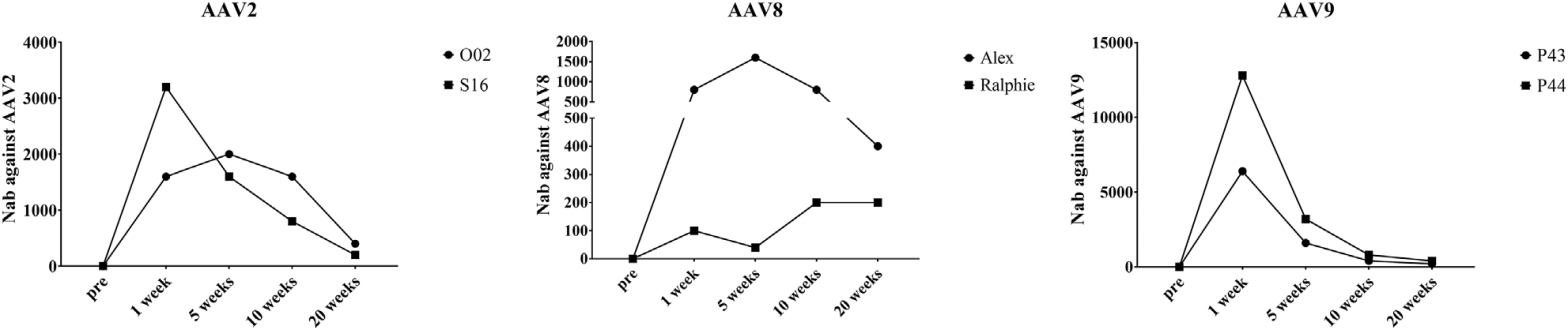
The Nabs against different AAV serotypes in canine plasma after AAV vectors administration. At different timepoints, plasma was obtained from the whole blood of hemophilia dogs that were injected with AAV2, AAV8 or AAV9 vectors at a dose of 1 × 10^12^vg/kg. AAV2, AAV8 or AAV9/luciferase vectors were incubated with a serial dilution of plasma. Then, transduction of the pre-incubated AAV2, AAV8, or AAV9/luciferase vectors with the plasma in Huh7 cells at 48 h s was measured *via* luciferase assay to evaluate the Nab levels.

Neutralizing antibodies (Nabs) to the AAV capsid imposes a barrier for gene therapy and the potential of re-administration. In this study, we also documented the Nabs evolution after AAV administration. In all six hemophilia dogs, there were no capsid specific antibodies before AAV injection. As shown in Supplementary Table S1, the Nabs in two hemophilia dogs treated with AAV2 peaked at 1 week (1:3,200) and 5 weeks (1:2,000), respectively, post AAV injection and gradually decreased to a relatively low level (1:400, 1:200) after 20 weeks (Figure 7). One AAV8 treated dog, Alex, peaked at 5 weeks after AAV injection (1:1,600); another dog treated with AAV8, Ralphie, remained at a relatively low level (1:40–1:200) during the 20 weeks (Figure 7) without a noteworthy peak. The Nab level induced by the AAV9 vectors in the hemophilia dogs, which was higher than that induced by AAV2 and AAV8, peeked at 1 week (1:6,400 and 1:12,800) post administration and decreased to a relatively low level (1:200, 1:400) at 20 weeks (Figure 7).

## Discussion

Considering the difference in AAV transduction efficacy between mice or other preclinical animals and clinical trials, a novel animal model that can predict AAV transduction in human is urgently needed. A chimeric mouse xenograft model with human cells has been developed to assess AAV transduction (Azuma et al., 2007; Bissig-Choisat et al., 2015). The immunodeficient mouse, like the FRG mouse, engrafted with human hepatocytes has been utilized to isolate human liver tropic AAV variants or evaluate AAV transduction efficiency in human hepatocytes (Lisowski et al., 2014; Li et al., 2015; Wang et al., 2015; Vercauteren et al., 2016; Shao et al., 2019). However, there has been insufficient evidence to demonstrate that the humanized chimeric mouse model can be used to reveal the actual transduction efficiency of AAV in human. Given the ethical concerns in comparing transduction efficiencies from different AAV serotypes or variants/mutants in humans and limited data that can be obtained from current clinical trials, in this study, we investigated whether AAV transduction in canine hepatocytes of chimeric mice could predict the result in real dogs. AAV gene therapy has been well studied in hemophilia, in which the liver is the main targeted organ for gene delivery. Therefore, hemophilia B animal models have been broadly utilized to explore the efficacy of AAV in the liver. Canine FIX can be secreted from the graft and murine FIX can be generated in canine hepatocyte xenograft mice. There is no available hemophilia mouse model with immunodeficiency for generation of chimeric hemophilia mice xenografted with canine hepatocytes. Moreover, the efficient AAV transduction involves many steps including binding to cell surface, endocytosis, viral trafficking in the cytoplasm, nuclear entry, uncoating, and second-strand synthesis. All of these steps are transgene independent. The transduction efficiency has been extensively studied in animal models with various transgenes in AAV vectors and consistent results have been achieved with one specific serotype. Therefore, we used GFP as a marker transgene to evaluate AAV transduction efficiency in chimeric mice and canine FIX to examine transduction efficacy in hemophilia B dogs between AAV serotypes. Herein, we first established the chimeric xenograft mice with canine hepatocytes and investigated the transduction efficiency of AAV2, AAV3, AAV5, AAV7, AAV8, and AAV9 in canine hepatocytes in the chimeric mice. Then, we evaluated the transduction efficacy of AAV vectors from three serotypes (AAV2, AAV8, and AAV9) in the liver in hemophilia dogs. We found that AAV8 was the best serotype to transduce canine hepatocytes in chimeric mice, followed by AAV9, then AAV3, 5, 7 and 2 with the lowest efficiency. Consistent with the results in chimeric mice, AAV8 induced the highest transgene cFIX expression followed by AAV9 and then AAV2 in hemophilia B dogs. These results suggest that chimeric mice may serve as a potential platform to predict AAV transduction in the liver in real species including large animals and humans.

It is well documented that the efficacy of AAV gene transfer varies between different animal models and humans (Nathwani et al., 2011a; Pavlou et al., 2021), although the mechanism remains to be clarified. Effective AAV vector transduction involves several steps including virion binding on the cell surface, virion uptake via endocytosis, escape from the endosome, traveling in the cytoplasm, and finally entrance into the nucleus and uncoating followed by transcription. The trafficking efficiency from endosome escape to the nuclear entrance in hepatocytes showed species specificity according to the study from Markusic et al., who found that a similar gene copy number in a primate model was detected but a lower mRNA level of the transgene was induced when compared to that in the murine model (Markusic et al., 2017). This study indicates AAV trafficking might be one of the reasons resulting in interspecies variation of AAV transduction.

In this study, we found that AAV8 induced the highest transduction in canine hepatocytes, followed by AAV9, and then AAV2 in chimeric mice xenografted with canine hepatocytes. In hemophilia B dogs, generally, the dogs treated with AAV8-cFIX vectors induced the highest cFIX expression and activity over AAV2 and AAV9. Both dogs with AAV9-cFIX vectors had higher cFIX expression and activity than those with AAV2. However, the data from dogs is inconsistent to that found in mice. AAV8 and AAV9 exhibited a similar efficacy in hemophilia B mice, which was also higher than AAV2. Though there has been no report about AAV transduction efficiency in canine hepatocytes of a chimeric mouse model, while a few studies have compared the transduction efficacy of different AAV vectors in dog and mouse models. Kazazian, et al. reported that both AAV8 and AAV9 induced a long-term transgene expression in hemophilia A dogs, but AAV8 induced a higher FVIII level in blood than AAV9 (Sarkar et al., 2006; Sabatino et al., 2011). In other studies, consistent with our results, AAV8 had a higher transgene expression than AAV2 in dogs (Jiang et al., 2006; Bell et al., 2011; Markusic et al., 2017). In mouse models, AAV8 has been demonstrated to transduce significantly more hepatocytes than AAV2 (Monahan et al., 2010). AAV8 transduction was over 10 times greater than AAV2 transduction in mouse liver (Gao et al., 2002; Zincarelli et al., 2008; Kattenhorn et al., 2016). No significant difference was found between AAV8 and AAV9 for liver transduction efficiency in both hemophilia A mice (Sarkar et al., 2006) and hemophilia B mice (Vandendriessche et al., 2007). When it comes to the wild-type mouse, it has been reported that AAV9 has a slightly greater transduction efficacy than AAV8 in the liver of wild-type mice (Inagaki et al., 2006; Bish et al., 2008; Zincarelli et al., 2008). It is unclear which mechanisms result in the different transduction efficiencies in the liver between wild type mice and hemophilia mice.

The immune response elicited against AAV vectors is one major concern for AAV gene therapy *in vivo*, including T-cell and B-cell responses. Currently one of the challenges for AAV gene therapy for patients is the existence of AAV neutralizing antibodies (Nabs) which are present in almost 50% of the human population, restricting patient eligibility for AAV mediated gene therapy. Additionally, after administration of AAV vectors, Nabs are induced, which make re-administration of AAV vectors impossible. It is interesting to note that the humoral immune response to the AAV capsid in human differs from that in animal models. In clinical trials for hemophilia B, the titer of neutralizing antibodies to AAV rose sharply in the first few weeks and then remained stable over the following 12 months or years (Nathwani et al., 2014). However, in pre-clinical research with hemophilia dogs, the Nab titer to AAV starts to drop a few weeks after AAV administration (Manno et al., 2006; Sun et al., 2018). It is also worth noting that the efficiency of Nab induction following AAV administration in dogs is different for the different serotypes. The Nab titer against AAV8 in both hemophilia B dogs after AAV8 vector administration displayed the lowest level among the three serotypes and the Nab titer in the dogs administered with AAV9 was much higher than that of AAV2 and AAV8. The direct mechanism that contributes to this discrepancy for Nab kinetics and titers between animal models and humans is currently unknown. The neutralizing antibodies to AAV capsid generated from AAV administration has also raised the concern for re-administration. Recently, numerous strategies have been proposed to tackle this obstacle for AAV gene therapy including IgG-cleaving endopeptidase treatment (Leborgne et al., 2020), development of novel AAV capsids (Paulk et al., 2018; Pekrun et al., 2019; Pei et al., 2020), and AAV empty virion acting as a decoy (Mingozzi et al., 2013; Gao et al., 2014).

In hemophilia clinical trials, the transgene expression was decreased at 6–10 weeks after AAV liver targeting in some patients. It has been suggested that capsid specific T cells can be activated and then eliminate AAV transduced hepatocytes leading to therapeutic failure. In this study, we did not detect any CTL response against capsids from AAV2, AAV8 or AAV9 in hemophilia dogs at any time points. Similar results were reported from another study showing a lack of T cell responses against AAV8 or AAV2 capsid in dogs after AAV administration, though a transgene specific T cell response was observed (Bell et al., 2011). The role of capsid specific CTLs in the elimination of AAV transduced cells needs further investigation.

A series of studies demonstrated that liver directed AAV gene therapy is able to induce systemic immune tolerance by exploiting the tolerogenic nature of the liver. We detected an increase of Tregs in the PBMCs of hemophilia B dogs receiving AAV8 vectors but did not observe that in the AAV2 and AAV9 treated dogs. This finding is consistent with previous studies that AAV8 vectors induce a stronger immune tolerance than other serotypes (Cooper et al., 2009; Bartolo et al., 2019). It was previously demonstrated that AAV8 is more potent for inducing an increase in Tregs than AAV2 (Cooper et al., 2009). It has been reported that intravenous injection of AAV8 vectors is able to induce immune tolerance and reverse cellular responses to the transgene via exhausting antigen-specific CD8^+^ T cells (Markusic et al., 2013; Bartolo et al., 2019). Recently, several studies in mouse and large animal models with hemophilia A demonstrated that AAV liver gene therapy could induce immune tolerance and prevent inhibitor development or even eradicate pre-existing FVIII inhibitors (Samelson-Jones and Arruda, 2020). The immune tolerance of AAV8-FVIII could be enhanced following B cell reconstitution in the hemophilia mouse model and might be used for immune tolerance induction therapy (Biswas et al., 2020). In hemophilia A gene therapy, almost all the mice injected with AAV/FVIII vectors could induce FVIII inhibitors. Our recent study also showed the development of FVIII inhibitors in one hemophilia A dog after re-administration of AAV8/hFVIII. Therefore, some caution should be taken when AAV liver targeted gene delivery is considered as an immune tolerance induction approach since the frequency of inhibitors in patients with hemophilia A is 15%–30% and only 3%–5% in patients with hemophilia B after protein replacement therapy (Perrin et al., 2019). Compared to AAV2 and AAV9, AAV8 induced a higher Treg and lower Nab titer after systemic administration in hemophilia dogs in this study. It is of clinical significance to study the role of Treg cells induced from AAV liver targeting in the Nab production after systemic administration of AAV vectors. The mechanism of immune tolerance induced from AAV liver targeting gene delivery is unclear. One study has demonstrated that both induction of Tregs and deletion of antigen specific T effector cells after AAV liver targeting may be dependent on the Fas/FasL pathway to induce immune tolerance (Keeler et al., 2019).

Since the chimeric mice lack T and B cells, an adaptive immune response couldn’t be elicited in these mice. Even if it is difficult to simulate adaptive immune response in this chimeric model, these chimeric mice could be used to study adoptive immunity after AAV application by adoptive transfer of canine PBMC cells (including T and B cell) to mimic adaptive immune response in dogs. However, this may give rise to GVHD (graft versus host disease) and impact observations of the immune response raised by gene therapy. Of course, the chimeric mouse model can be used to study the innate immune response in AAV transduced canine hepatocytes. We have used human hepatocyte xenografted mice to study the innate immune response activation in AAV transduced human hepatocytes (Shao et al., 2018).

Though our data indicated that chimeric mice xenografted with hepatocytes from human liver could be a useful tool to evaluate relative performance of different AAV vectors and isolate novel AAV capsids in human gene therapy, challenges still remain. One of them is the individual variation in xenografted hepatocytes, which contributes to the differences in the group within the same donor. We have observed differences in transduction efficiency in canine hepatocytes of chimeric mice administrated with the same serotype of AAV vectors (for example, 11.58% and 2.15% for two individual mice in the AAV5 group). Moreover, the transduction efficiency varies in the liver of chimeric mice xenografted with hepatocytes from different donors even though the same dose of AAV vectors was used. The transduction efficiency of AAV8 vectors in mice engrafted canine hepatocytes from donor #2 (25.71% and 30.73%) was higher than that from donor #1 (12.65%, 11.49%, and 30.73%). The genetic background among different individuals and engraftment efficiency of canine hepatocytes might contribute to the transduction variations of AAV serotypes, which suggest that chimeric mice xenografted with hepatocytes from more individuals should be included in future studies. Although the data from chimeric mice xenografted with canine hepatocytes can be used to predict the actual transduction efficiency in the liver of dogs, more studies in chimeric mice xenografted with hepatocytes from other species are still warranted to prove the value of chimeric mice xenografted with human hepatocytes in future AAV clinical studies.

## Data Availability

The original contributions presented in the study are included in the article/Supplementary Material, further inquiries can be directed to the corresponding author.
