# Supplementary material for: Chimeric Mice Engrafted With Canine Hepatocytes Exhibits Similar AAV Transduction Efficiency to Hemophilia B Dog
Source: Front Pharmacol. 2022 Jan 31;13:815317. doi: 10.3389/fphar.2022.815317 (PMC8841897; doi:10.3389/fphar.2022.815317)
Supplement: Supplementary file 1 [file DataSheet1.PDF]

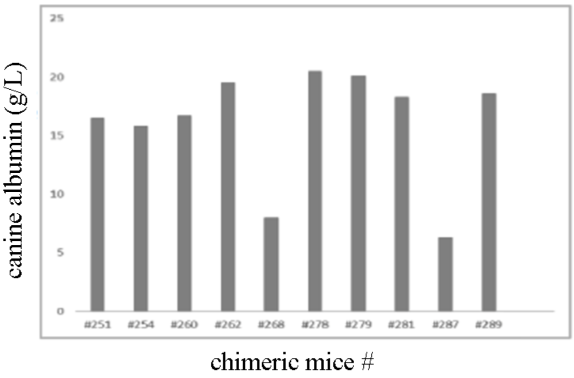

Supplementary Figure 1. Canine albumin detection in chimeric mice serum. Serum Canine albumin in 10 chimeric mice was detected by ELISA at week 9 after transplantation of canine hepatocytes.

A

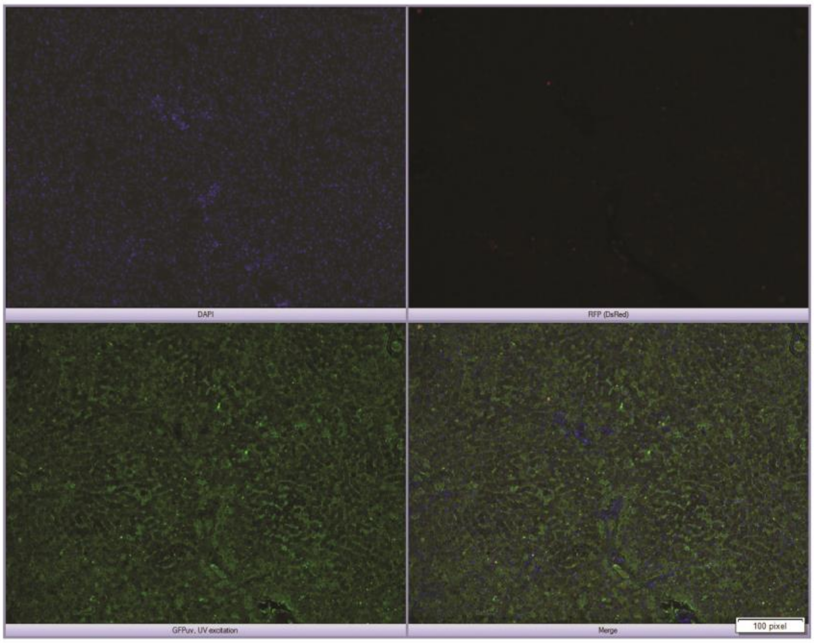

B

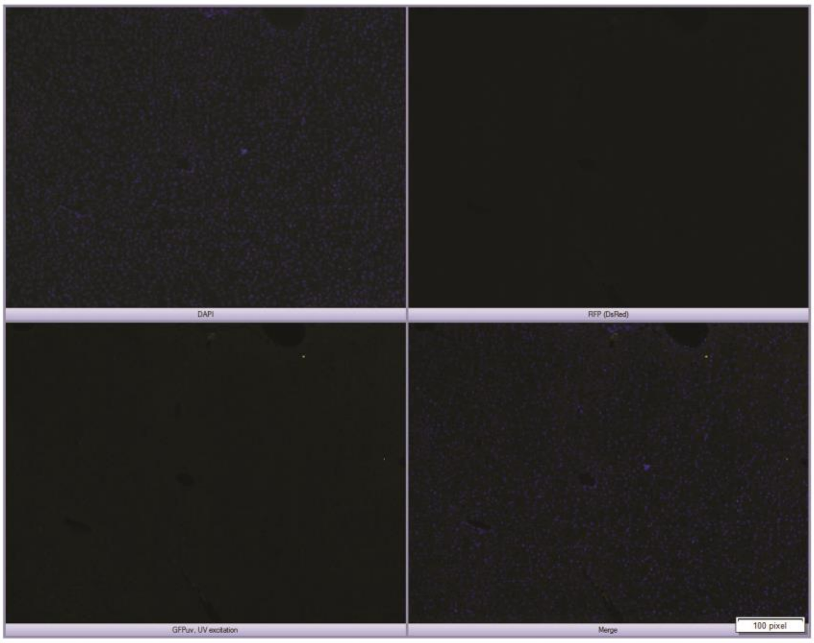

Supplementary Figure 2. Wild type dog (**A**) and mouse (**B**) liver staining. Wild type dog and mouse liver tissue sections were harvested and stained the same as the xenograft mouse liver tissues. The canine hepatocytes were stained green by an anti-canine albumin antibody (bottom left). And GFP protein was stained red by an anti-GFP antibody (upper right). The cell nucleus was stained by DAPI (upper left). The merged image is shown in the bottom right.

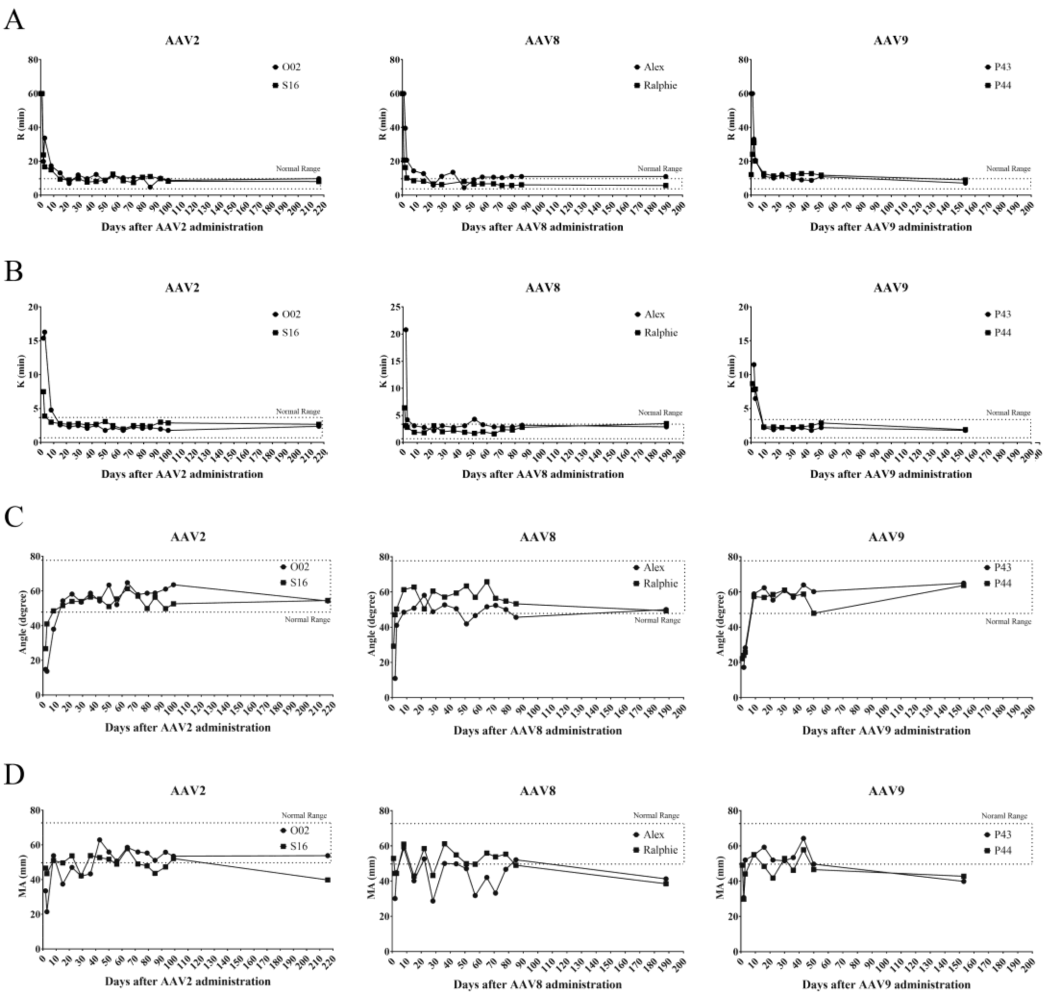

Supplementary Figure 3. Thromboelastography analysis after AAV-cFIX administration. Thromboelastometry assay was performed at the different time points after administration of AAV2, AAV8 or AAV9 vectors packaged with cFIX-opt-R338L transgene in hemophilia B dogs. **A,** The reaction time in minutes represents the time to initial fibrin formation. **B,** The kinetics time in minutes represents the time to clot formation. **C,** The α angle in degrees represents the rate of clot formation. **D,** Maximum clot firmness in mm represents the absolute clot strength. Normal ranges for each parameter is represented in the dashed box.

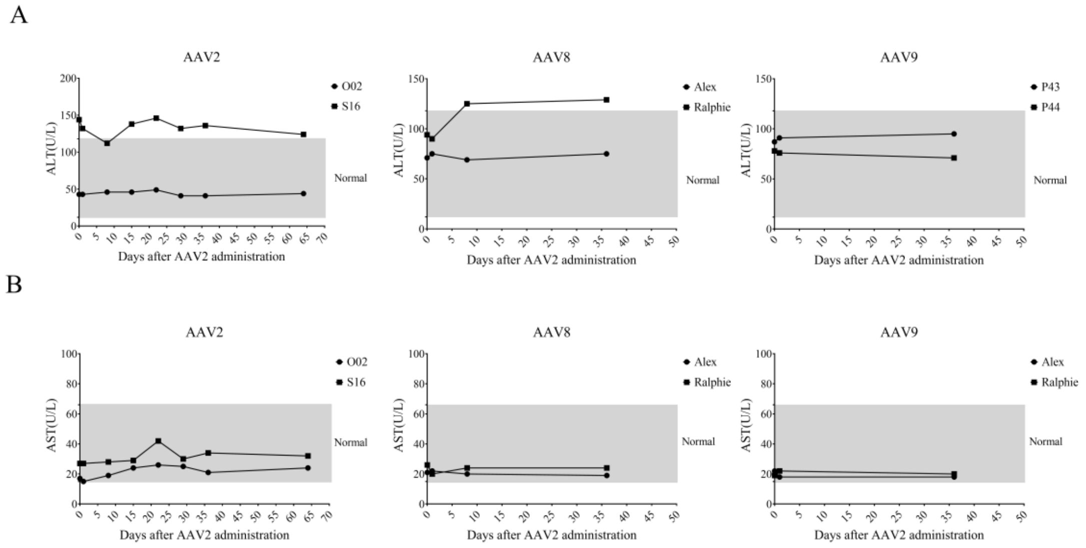

Supplementary Figure 4. Liver Enzyme detection after AAV8-cFIX administration. (**A**) Serum alanine transaminase (ALT) and (**B**) aspartate transaminase (AST) were monitored at different time points in hemophilia B dogs treated with AAV2, AAV8 and AAV9 vectors encoding cFIX-opt-R338L. The normal range of canine ALT is 12–118 U/L and a normal AST level is 15–66 U/L, which is indicated by the gray area.

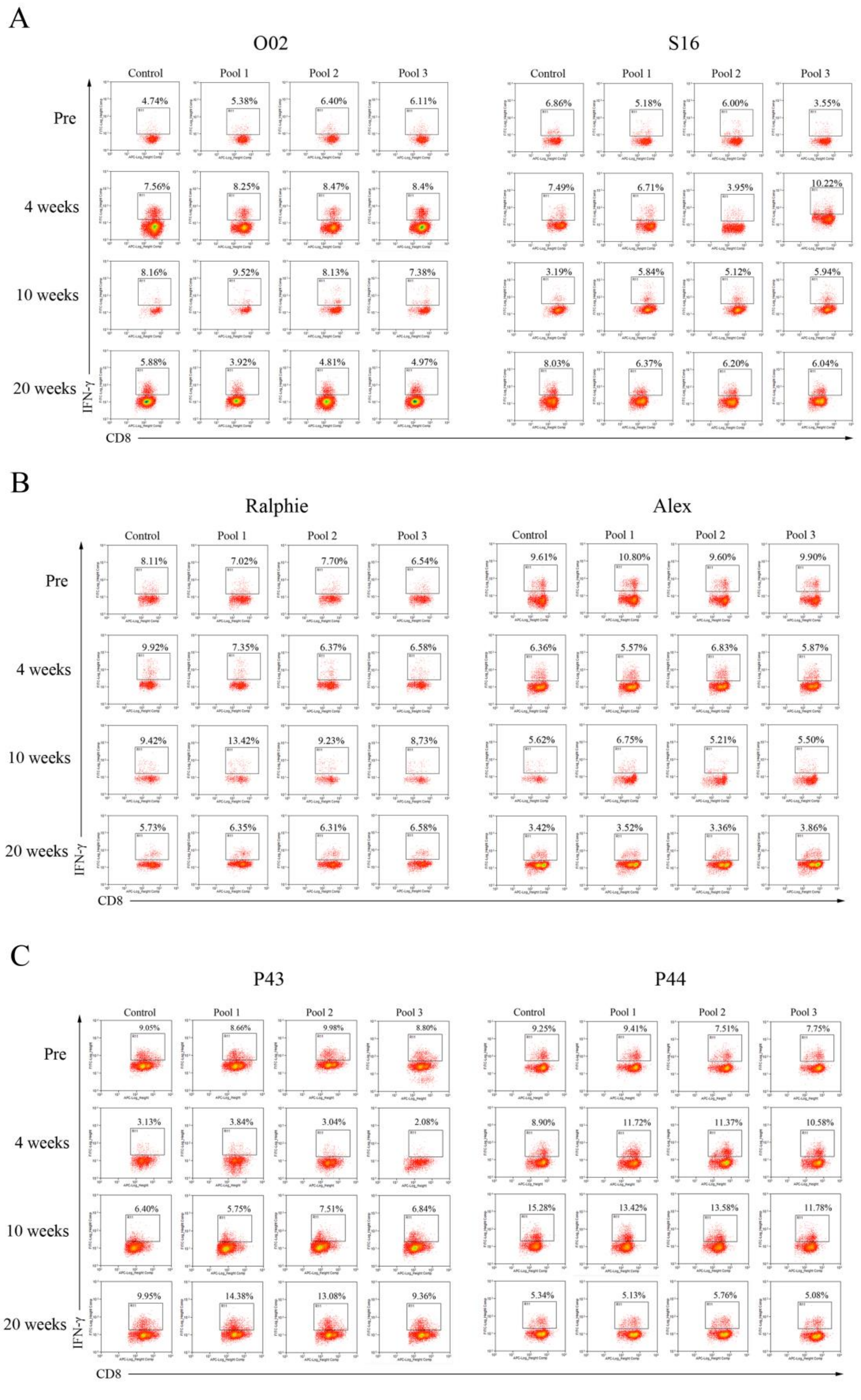

Supplementary Figure 5. Capsid specific CTL detection after administration of

varying AAV/cFIX vector serotypes. PBMCs separated from the whole blood of hemophilia dogs injected with AAV2/cFIX (**A**), AAV8/cFIX (**B**) or AAV9/cFIX (**C**) were cultured in the presence of 3 different AAV2 capsid peptide pools for 48hrs. Then the PBMCs were double stained with CD8 and IFN-γ antibodies and analyzed by Flow Cytometry. After gating of $CD8^+$ cells, the percentage of IFN-γ^+ cells was shown.

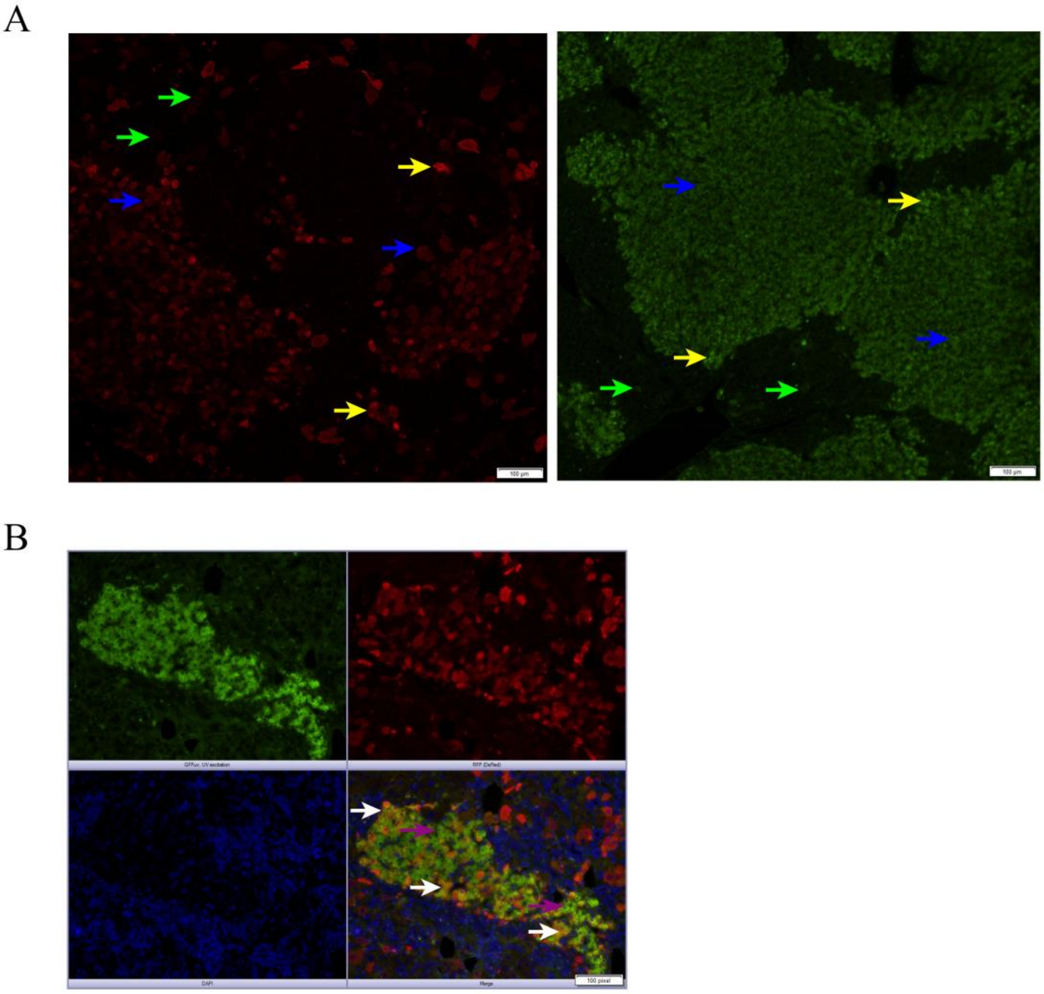

Supplementary Figure 6. Representative image to show low, medium, or high staining cells and the GFP^+ canine $albumin^+$ cells. A, Representative low (green), medium (blue), or high (yellow) staining cells for GFP or albumin was pointed by

different colored arrows. **B,** the cells showing co-localization of canine albumin and GFP was indicated by white arrows, while untransduced canine hepatocytes was indicated out by purple arrows.

Supplementary Table 1. The Nab titers against different AAV serotypes in canine plasma after AAV vectors administration

Group	AAV2		AAV8		AAV9	
	O02	S16	Alex	Ralphie	P43	P44
Peak Time (weeks)	5	1	5	10-20	1	1
Peak Nab Titer	1:2000	1:3200	1:1600	1:200	1:6400	1:12800
Nab Titer at 20 weeks	1:400	1:200	1:400	1:200	1:200	1:400
